# Exogenous Restoration of TUSC2 Expression Induces Responsiveness to Erlotinib in Wildtype Epidermal Growth Factor Receptor (EGFR) Lung Cancer Cells through Context Specific Pathways Resulting in Enhanced Therapeutic Efficacy

**DOI:** 10.1371/journal.pone.0123967

**Published:** 2015-06-08

**Authors:** Bingbing Dai, Shaoyu Yan, Humberto Lara-Guerra, Hiroyuki Kawashima, Ryo Sakai, Gitanjali Jayachandran, Mourad Majidi, Reza Mehran, Jing Wang, B. Nebiyou Bekele, Veerabhadran Baladandayuthapani, Suk-Young Yoo, Ying Wang, Jun Ying, Feng Meng, Lin Ji, Jack A. Roth

**Affiliations:** 1 Section of Thoracic Molecular Oncology, Department of Thoracic and Cardiovascular Surgery, University of Texas (UT) MD Anderson Cancer Center, Houston, Texas, United States of America; 2 Department of Biostatistics, UT MD Anderson Cancer Center, Houston, Texas, United States of America; 3 Department of Bioinfomatics and Computational Biology, UT MD Anderson Cancer Center, Houston, Texas, United States of America; 4 Nigata University Graduate School of Medical and Dental Sciences, Nigata, Japan; 5 Okayama Citizens’ Hospital, Okayama, Japan; 6 Gilead Sciences, Foster City, California, United States of America; Penn State Hershey Cancer Institute, UNITED STATES

## Abstract

Expression of the tumor suppressor gene TUSC2 is reduced or absent in most lung cancers and is associated with worse overall survival. In this study, we restored TUSC2 gene expression in several wild type EGFR non-small cell lung cancer (NSCLC) cell lines resistant to the epidermal growth factor receptor (EGFR) tyrosine kinase inhibitor erlotinib and analyzed their sensitivity to erlotinib in vitro and in vivo. A significant inhibition of cell growth and colony formation was observed with TUSC2 transient and stable expression. TUSC2-erlotinib cooperativity in vitro could be reproduced in vivo in subcutaneous tumor growth and lung metastasis formation lung cancer xenograft mouse models. Combination treatment with intravenous TUSC2 nanovesicles and erlotinib synergistically inhibited tumor growth and metastasis, and increased apoptotic activity. High-throughput qRT-PCR array analysis enabling multi-parallel expression profile analysis of eighty six receptor and non-receptor tyrosine kinase genes revealed a significant decrease of FGFR2 expression level, suggesting a potential role of FGFR2 in TUSC2-enhanced sensitivity to erlotinib. Western blots showed inhibition of FGFR2 by TUSC2 transient transfection, and marked increase of PARP, an apoptotic marker, cleavage level after TUSC2-erlotinb combined treatment. Suppression of FGFR2 by AZD4547 or gene knockdown enhanced sensitivity to erlotinib in some but not all tested cell lines. TUSC2 inhibits mTOR activation and the latter cell lines were responsive to the mTOR inhibitor rapamycin combined with erlotinib. These results suggest that TUSC2 restoration in wild type EGFR NSCLC may overcome erlotinib resistance, and identify FGFR2 and mTOR as critical regulators of this activity in varying cellular contexts. The therapeutic activity of TUSC2 could extend the use of erlotinib to lung cancer patients with wildtype EGFR.

## Introduction

The systemic delivery of transcriptionally active tumor suppressor genes to cancer cells can potentially address the problem of undruggable mutated tumor suppressor genes which are the most common genetic abnormalities found in cancer. We recently reported systemic delivery of TUSC2 (also known as FUS1) gene at therapeutically active levels to disseminated lung cancer using a non-immunogenic nanovesicle [1]. Allelic loss of the 3p chromosome, in particular the 3p21.3 region, which harbors several tumor suppressor genes, frequently occurs in lung and other cancers [2–5]. TUSC2 is one of the tumor suppressor genes in this region that has been extensively characterized [6–8]. We reported that TUSC2 restoration in 3p21.3-deficient non-small cell lung cancer (NSCLC) cells suppressed tumor growth by induction of apoptosis and alteration of cell kinetics in vitro and in vivo through Apaf-1 [9]. In addition, evidence indicated that TUSC2 downregulates the activation of numerous tyrosine kinases, including EGFR [10,11].

Erlotinib (Tarceva) (N-(3-ethynylphenyl)-6, 7-bis(2-methoxyethoxy)4-quinazolinamine) is an orally active selective inhibitor of the epidermal growth factor receptor (EGFR) tyrosine kinase [12]. It has been used clinically for the treatment of advanced lung cancer [13]. However, resistance rapidly develops in patients who relapse after initial response to EGFR tyrosine kinase inhibitor therapy [14,15]. Erlotinib exerts antitumor activity through inhibition of EGFR tyrosine kinase, but its antitumor activity is not correlated with the level of EGFR expression by tumor cells. Differences in the presence of activating mutations in the EGFR gene between erlotinib responders and non-responders have been reported, and these EGFR mutations seem to be predictive markers for sensitivity to erlotinib and gefitinib (Iressa, N-(3-chloro-4-fluoro-phenyl)-7-methoxy-6-(3-morpholin-4-ylpropoxy)quinazolin-4-amine), another EGFR tyrosine kinase inhibitor [16–18]. Although some delay in tumor progression has been noted in patients with wildtype EGFR, this population is generally unresponsive to erlotinib.

Considering the increased use of erlotinib in EGFR mutant NSCLC patients, the question of its benefits in patients without EGFR mutations has recently gained attention. In this report, we hypothesized that the combination of TUSC2 gene restoration and erlotinib treatment could enhance antitumor therapeutic efficacy in wild type EGFR NSCLC cells, characterized as erlotinib-resistant. We provide in vitro and in vivo evidence for TUSC2-erlotinib cooperativity in vitro and in vivo. The ability of TUSC2 to sensitize wild type EGFR NSCLC cells to erlotinib was tested in a several cell lines and two mouse models. We show a significant increase in apoptotic activity and identify FGFR2 and mTOR as critical regulators of this cooperative effect.

## Material and Methods

### Cell lines and Cultures

The human NSCLC cell lines A549, H322, H460, and H1299 were provided by Dr. John V. Heymach (MD Anderson Cancer Center) and Drs. Adi Gazdar and John D. Minna (The University of Texas Southwestern Medical Center at Dallas). The human NSCLC cell line H157 was obtained from American Type Culture Collection (Manassas, VA). Cells were maintained in RPMI-1640 medium, supplemented with 10% heat-inactivated fetal bovine serum, 1% Glutamine, 1% penicillin, and 1% streptomycin. All cells are wild type EGFR and have low or absent expression level of TUSC2 protein.

### Reagents

Erlotinib; AZD4547 and Rapamycin; FGFR2, PARP, and mTOR (Ser2448) antibodies were purchased form Pfizer (NY, NY), Selleckchem (Houston, TX), and Cell Signaling Technology (Danvers, MA), respectively. TUSC2 poylclonal antibody was developed in Bethyl Laboratories, Montgomery, TX. DOTAP and cholesterol were purchased from Avanti Polar Lipids (Albaster, AL). Lipofectamine 2000 was purchased from Invitrogen Corporation (Carlsbad, CA). FGFR2 siRNA was purchased from Santa Cruz Biotechnology (Santa Cruz, CA). DOTAP:cholesterol (DC)–TUSC2 complexes were made as previously described [1].

### Establishment of Tet-inducible TUSC2 Expressing Cell Lines

Tet-inducible TUSC2 expressing cells were made using Lenti-X Tet-On advanced inducible expression system (Clontech) according to the manufacturer’s instructions. Clones were maintained in RPMI-1640 medium, supplemented with 10% Tet-free heat-inactivated fetal bovine serum (Clontech). Briefly, A549, H1299, and H157 cells were infected with lentivirus generated with the pLVX-Tet-On advanced vector to constitutively express the tetracycline-controlled transactivator. After G418 selection, surviving colonies were expanded and infected with pLVX-Tight-Puro-Luc. New colonies were selected and expanded under G418 and puromycin. Best functional transactivators were selected after exposing cells to 2μg/ml of doxycycline for 48 hrs. Luciferase activity in cell extracts was assessed using Promega Luciferase Assay (Promega). Clones were assessed for TUSC2 expression induction by Western blotting.

### Transient Transfection and RNA Interference Induction

TUSC2 transient transfection was carried out with 4μg TUSC2 using Lipofectamine 2000, and optimal transfection efficiency was assessed with green fluorescent protein (GFP). Cells were transfected with 50nM FGFR2-specific siRNA to suppress FGFR2 gene expression, which was confirmed by Western blotting.

### Cell Viability Assay

Sulforhodamine B (SRB) assay (Sigma-Aldrich) was used to assess tumor cell viability on the following: 1) TUSC2-inducible stable clones, A549, H1299, and H157 cells, treated with 1μg/ml doxycycline for 24 hours followed by exposure to 2.3μM erlotinib for 48 hours. Untreated cells or cells treated with either doxycycline or erlotinib, alone, served as controls. 2) H1299, A549, H322, and H460 cells treated with 0.1μM or 1.0μM AZD4547, selective FGFR2 inhibitor, alone or with varying concentrations of erlotinib, ranging from 0.01 to 10μM. Untreated cells and cells treated with erlotinib alone were used as a controls. 3) A549 cell transfected with FGFR2-siRNA construct and treated with varying concentrations of erlotinib, ranging from 0.01 to 10μM. Cells treated with erlotinib alone (Mock) or cells transfected with siRNA without the FGFR2 construct and treated with erlotinib were used as controls. 4) H1299, H322, and H460 cells treated with 1μM or 0.5μM Rapamycin, mTOR selective inhibitor, alone, or with varying concentrations of erlotinib, ranging from 0.01 to 10μM. Cell viability was quantified after 48 hours. Each treatment contained 24 observations obtained from 3 batches of experiments. The percentage of viable cells was determined by the ratio of absorbance of treatment and control groups: ODT/ODC x 100%. Each treatment contained 24 observations obtained from 3 batches of experiments. Univariate analysis was performed to evaluate the distribution of data for each treatment group. To determine whether SRB % was different between treatment groups, three methods were used: 1) Assuming homogeneity among the three batches of experiments, ANOVA was performed to compare the variance between treatment groups for all samples within each cell line. Tukey’s multiple comparisons test was performed for pairwise differences between treatment groups; 2) Taking into consideration of potential random effect among experiment batches, mixed linear models were used to compare between treatment groups within each cell line. Tukey’s multiple comparisons test was performed for pairwise differences between treatment groups; 3) To assess the overall effect of doxycycline among all cell lines, a mixed linear model analysis was performed by using treatment and cell lines as fixed effect and batch as random effect. Multiple comparisons were adjusted using the Tukey method. P<0.05 was considered statistically significant; all tests were two-sided. Analyses were performed using SAS 9.3 (SAS Institute Inc., Cary, NC).

### Colony Formation Assay

Cells were co-transfected with 4μg TUSC2-pcDNA3 and 1μg of the neomycin-resistant gene pcDNA3 plasmids, and grown in medium containing 400μg/ml G418 prior to treatment with 1μM or 2.3μM erlotinib. Colonies were fixed with glutaraldehyde, stained with crystal violet, counted with a stereomicroscope, and analyzed with Image-J software. Values represent the mean of three independent experiments. The statistical significance of differences between TUSC2, erlotinib, and TUSC2 plus erlotinib treatments was calculated by two-tailed t test analysis; P< 0.05 was considered significant. The cooperative effect of DC-TUSC2 and erlotinib combination was assessed using a Bayesian parametric bootstrapping approach. Cooperative effects were determined using Pr (min (μF, μE) < μC |data). The Statistical software S-PLUS 8.0 was used for all analyses.

### H322 Subcutaneous Xenograft Mouse Model

Protocols were approved by the Committee on the Ethics of Animal Experiments of The University of Texas MD Anderson Cancer Center (Permit Number: 11-03-09933) in accordance with NIH Guidelines for the Care and Use of Laboratory Animals. Six to eight week old female mice were purchased from Charles River Laboratories (Wilmington, MA). Wild type EGFR, TUSC2-deficient human H322 cells (3×10^6^), and when tumors reached an average volume of about 0.1cm^3^, mice were randomly divided into five treatment groups (n = 9 animals per group): PBS; DC–TUSC2 plasmid complex; erlotinib; erlotinib plus DC–EV DNA complex; and erlotinib plus DC–TUSC2 complex. Animals received random intravenous injections with DC-TUSC2 complexes at a dose of 25μg of plasmid DNA and 10nmol DC in 100μL of 5% dextrose in water. Erlotinib was oral fed, 30mg/kg daily for eight days. CO2 was used to euthanize animals.

### Analysis of Tumor Growth

Tumor size and colony count assessments were made without knowledge of the treatment groups, and data was analyzed independently by the biostatistical group. Tumor volumes were determined every three days by measuring the longest diameter across the tumor and its corresponding perpendicular diameter(Length × Width2 × 0.52). The ratio of tumor sizes between treatment and control groups was used to evaluate tumor growth inhibition Generalized linear regression models were used to study tumor growth over time. A heterogeneous autoregressive covariance structure was used to account for inter-mouse variability and longitudinal nature of the data. An interaction between treatment and time was assessed to test the heterogeneity of slopes. CONTRAST statement in PROC MIXED procedure in SAS was used to compare tumor growth rates between each pair of the treatment groups. Transformation of logarithm to the base two of tumor size was used in the analyses to satisfy the normality assumption of the models. Co-operative effects of the combination were assessed using a Bayesian bootstrapping approach, and P< 0.05 was considered significant.

### A549 Xenograft Mouse Model of Metastatic Lung Cancer

Mice were injected, via tail vein, with 10^6^ luciferase-expressing human A549 tumor cells suspended in 200 ml of sterile PBS. When average size of tumors reached 5–8 mm in diameter (16 days), mice were randomly divided into the same five treatment groups as their subcutaneous counterparts (n = 9 animals per group). TUSC2 intravenous injection with systemic DC–based nanovesicles was used at a dose of 25μg of plasmid DNA and 10nmol DC. Erlotinib was given orally at a dose of 30mg/kg daily for eight days. Eight hours after the last dose of erlotinib and 21 days following the first treatment, animals were euthanized by CO2 inhalation.

### Analysis of Tumor Growth and Metastasis

Lungs were injected intratracheally with India ink, fixed in Feketes solution, and dissected lungs were analyzed by counting metastatic tumor nodules with a Nikon TC200 fluorescence microscope. All counts were repeated at least three times without knowledge of the treatment groups and the statistical significance of differences of tumor nodule counts between and within groups was analyzed by Wilcoxon rank-sum and Kruskal-Wallis tests; P< 0.05 was considered significant.

### In Situ Detection of Apoptosis

Apoptosis was determined in resected animal tumor tissue sections by TUNEL assay. Tumor specimens were washed with xylene, ethanol, and PBS. Apoptosis was assessed by labeling DNA strand breaks using ApopTag in situ apoptotic detection kit (Oncor, Gaithersburg, MD). Apoptotic cells were quantified and expressed as a percentage of the total number of cells. Values represent percentages from at least 1000 cells. The statistical significance of differences in apoptotic cells within and between the treatment groups was analyzed by Fisher's exact test. P< 0.05 was considered significant.

### RNA Purification

Total RNA was isolated using the RNeasy mini kit (Qiagen, Santa Clarita, CA) according to the manufacturer's instruction. Purity of the isolated total RNA was measured using Nano-drop and PCR with β-actin primers. Only RNA with an A260/A280 ratio >1.9 and no detectable contamination of DNA (PCR) was employed for qRT-PCR.

### Tyrosine Kinase qRT-PCR Array Screening

Template cDNAs were reverse transcribed from total RNA using the First Strand cDNA Synthesis Kit (SABiosciences, Frederick, MD). Changes in levels of 86 receptor and non-receptor tyrosine kinase-related genes were quantified using RT^2^ Profiler PCR Array, Applied Biosystems, Rockville, MD (S1 Table). Data were normalized by subtracting Ct values of “GAPDH" from Ct values of the 86 genes. The resultant ΔCt values were combined to calculate the average fold change values. A two-way ANOVA was performed to identify expressed genes that were significantly differentiated between control and treatment groups. A beta-uniform mixture (BUM) model was applied to adjust for multiple testing. Statistical analysis was performed using R packages, publicly available statistical computing software.

### Western Blot Analyses

Lysates form cells exposed to different treatments were quantified by Lowry assay (DC Protein assay, Bio-Rad, Hercules, CA). Equal amounts of protein were loaded onto SDS-PAGE, transferred to nitrocellulose membranes, immunoblotted with the primary and horseradish peroxidase-conjugated antibodies. Proteins were detected by chemiluminescence.

## Results

### TUSC2-Erlotinib Combination Significantly Inhibits Tumor Cell Viability and Colony Formation

**Fig 1:**
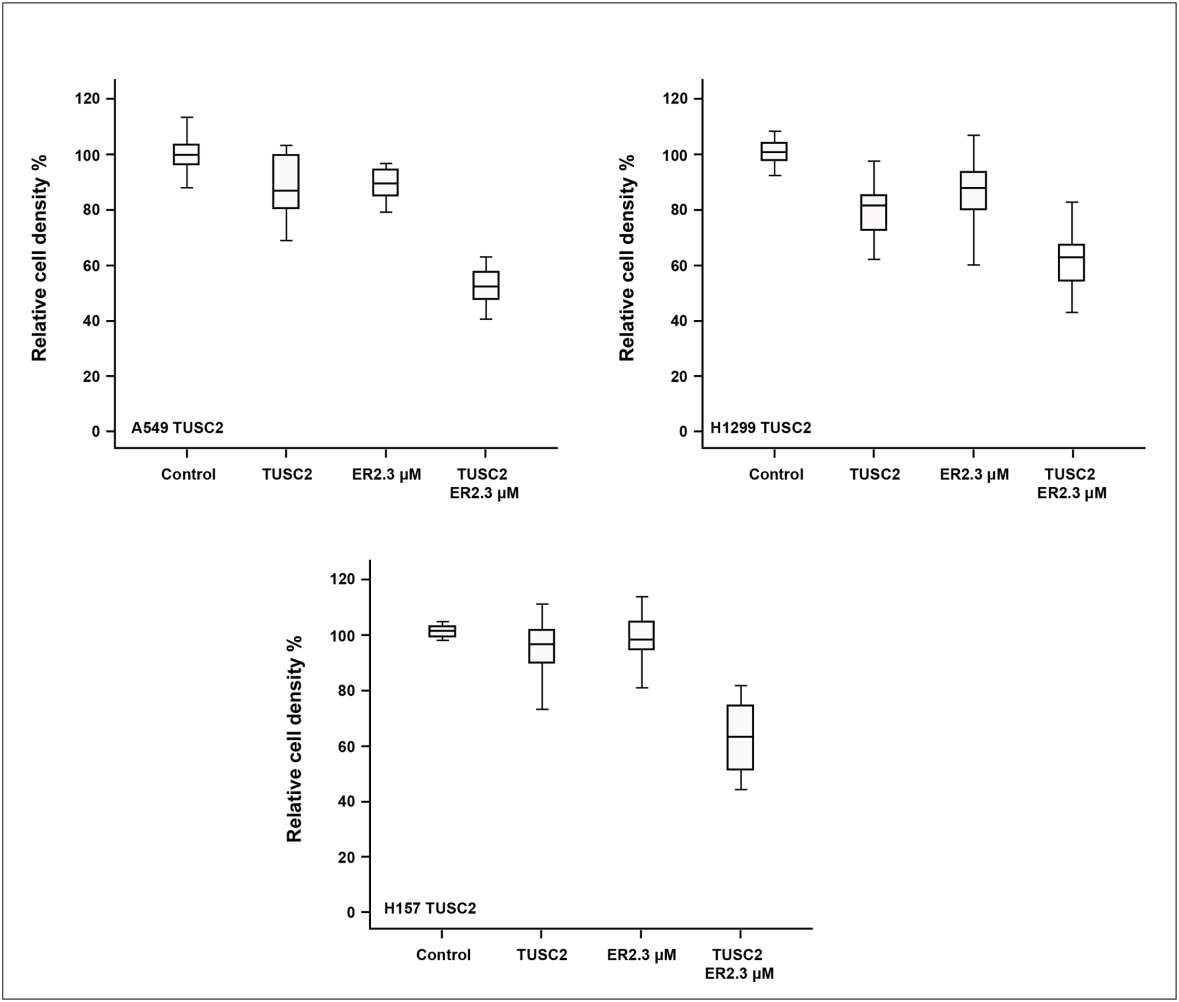
Combination of TUSC2 stable expression and erlotinib treatment inhibits tumor cell growth significantly. TUSC2-deficient wild type EGFR NSCLC A549, H1299, and H157 cells were treated with 1μg/ml doxycycline to induce TUSC2 expression, treated with 2.3μM erlotinib, and assayed for viability using the SRB assay. After adjusting for multiple comparisons, a significant inhibitory effect was observed for TUSC2 and erlotinib combined treatment compared to any other group by ANOVA (P<0.0001). Data shown is expressed as the mean ± SE. Values represent the mean of three independent experiments.

**Fig 2:**
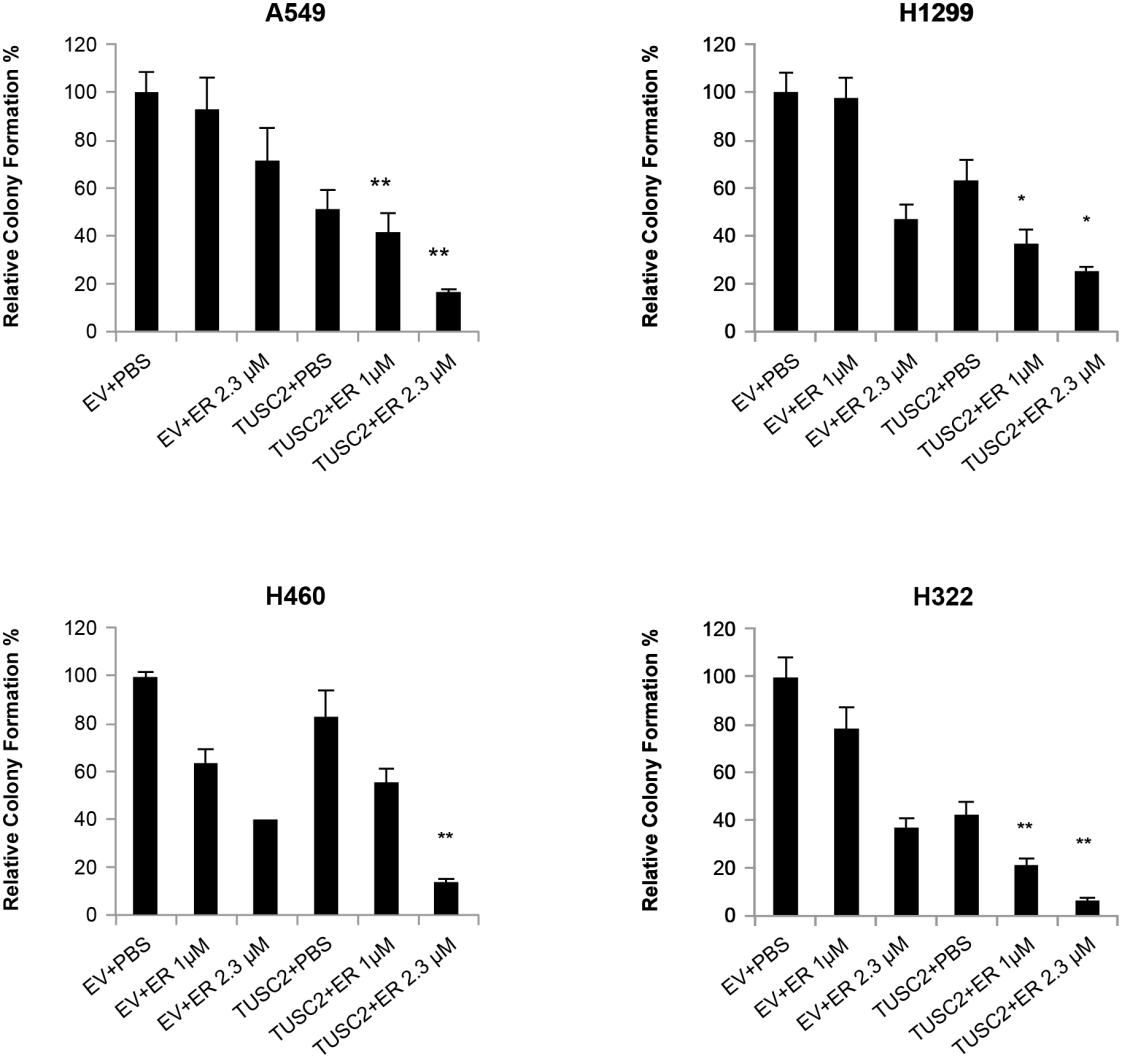
Combination of TUSC2 transient expression and erlotinib treatment inhibits colony formation significantly. TUSC2-deficient wild type EGFR NSCLC A549, H1299, H460, and H322, were transfected with four micrograms TUSC2 pcDNA3. After G418 selection, cells were treated with 1.0μM or 2.3μM erlotinib, colonies were fixed with glutaraldehyde, stained with crystal violet, and counted using a stereomicroscope. Data shown is expressed as the mean ± SE. Values represent the mean of three independent experiments. The statistical significance of differences was calculated by two-tailed t test analysis; *, P< 0.05, **, P<0.01. The cooperative effect of the TUSC2 transfection and erlotinib combination was >0.99 for each cell line.

A group of TUSC2-deficient wild type EGFR NSCLC lines were tested for sensitivity to erlotinib after restoration of TUSC2 expression, both transiently and stably (Table 1). We found a significant benefit of the combination at micromolar ranges between 1uM and 2.3uM. Because we were analyzing efficacy on a spectrum of biological processes, not dependent on the mutated EGFR target kinase, these concentrations which are achievable in patient serum with standard dosing regimens are pharmacologically relevant. Cell viability was evaluated in three TUSC2 Tet-On stable clones treated with doxycycline, to induce TUSC2, and combined with erlotinib. As expected, cells were not responsive to erlotinib alone. Viability of A549, H1299, and H175 was still 92%, 90%, and 98%, respectively. Induction of TUSC2 with doxycycline showed more cytoxicity than erlotinib, resulting in 16%, 22%, and 5% cell death, respectively (Fig 1). However, when cells were exposed to doxycycline and treated with 2.3 μM erlotinib for 48 hours, a significant growth inhibitory effect was observed for all three cell lines (p<0.05). The relative survival of A549, H1299, and H175 were reduced by 48%, 42%, and 38%, respectively. Similarly, colony formation was significantly inhibited in cells transiently transfected with TUSC2 and treated with erlotinib. The ability of A549, H1299, H322, and H460 cells to form colonies was reduced by 90%, 80%, 93%, and 85%, respectively (Fig 2). In dose titration experiments erlotinib mediated increased inhibition of colony formation at nanomolar concentrations (data not shown).

**Table 1:** Cell type and EGFR status in tested NSCLC cell lines.

Cell line	Tumor Subtype	EGFR Status
H322	Bronchoalveolar Carcinoma	WT
A549	Adenocarcinoma	WT
H1299	Adenocarcinoma	WT
H460	Large Cell Carcinoma	WT
H157	Squamous Cell Carcinoma	WT

Taken together, the results clearly demonstrate the superiority of the TUSC2-erlotinib combination treatment over each agent alone, and indicate that the effect is independent of the technique of exogenous gene expression. For both, viability and colony formation assays, the probability of a cooperative effect was greater than 0.99, on a scale from 0 to 1. Zero means no probability of a true cooperative effect, and one means 100% probability of a cooperative effect given the observed data.

### TUSC2-Erlotinib Combination Significantly Inhibits Tumor Growth and Metastasis and Induces Apoptotic Activity

**Fig 3:**
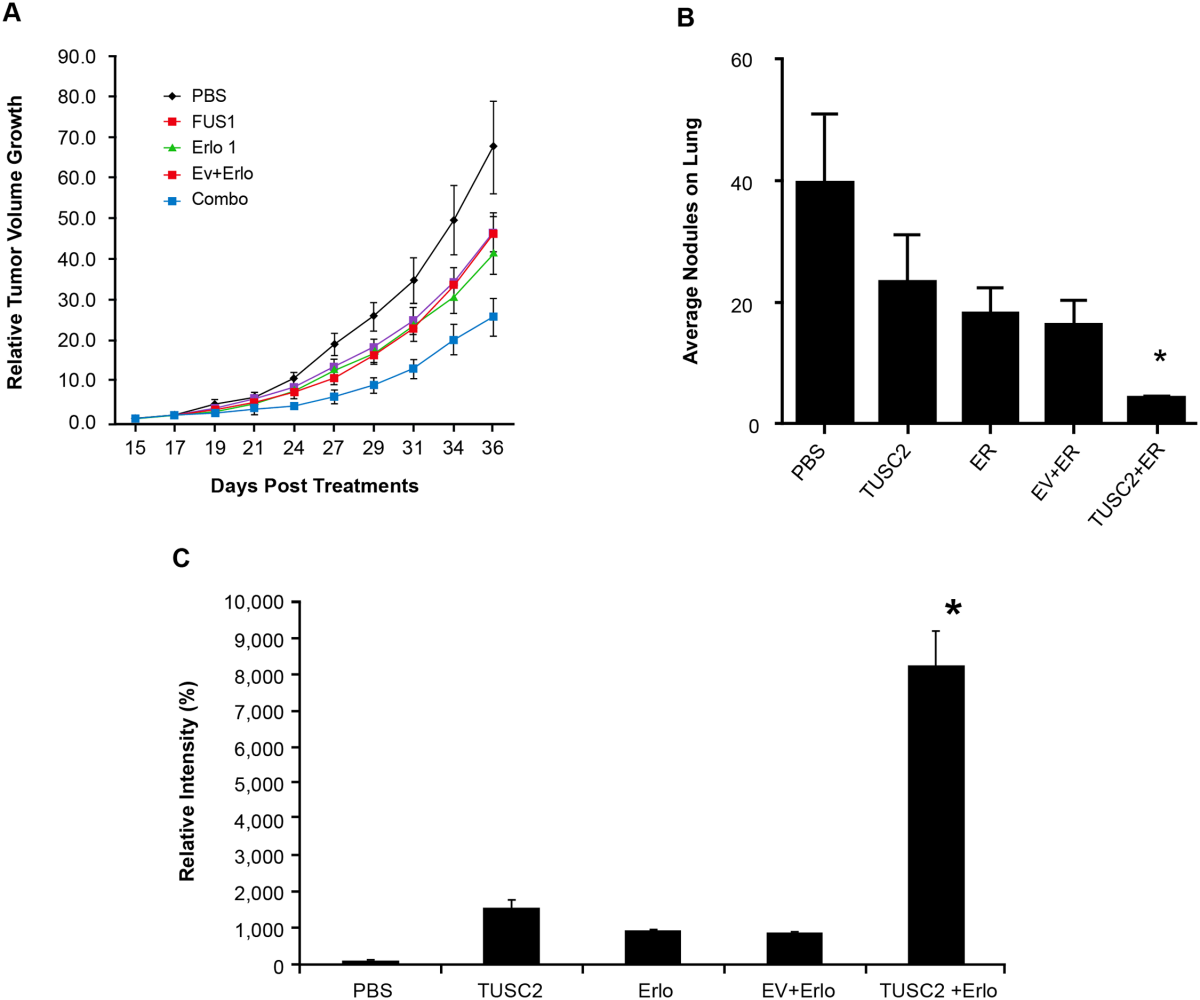
DC-TUSC2 nanovesicles and erlotinib combination treatment inhibits tumor growth and metastases in H322 subcutaneous (A), and A549 metastatic (B) xenograft mouse models. A) The change of tumor size over time (tumor growth rate or the slope of the linear regression model) differed by treatment (P<0.02 for the interaction between treatment and time). With adjustment for multiple comparisons, the tumor growth rate of the DC-TUSC2 and erlotinib combination group was the only group significantly smaller than the PBS group (P<0.01). The probability of a co-operative effect between DC-TUSC2 and erlotinib was > 0.99; B) Total tumor nodule counts on the lung surface are shown. Wilcoxon rank-sum and Kruskal-Wallis tests were used. The overall difference of the tumor nodule count among the five groups was significant (P<0.0001). The difference between the DC-TUSC2 and erlotinib combination group compared to each of the other groups was significant P<0.01. The probability of a cooperative effect of the DC-TUSC2 and erlotinib combination was >0.99. (C) Resected tumor tissues were assayed by TUNEL. Values represent percentages from at least 1000 counted cells. Fisher's exact test was used. *P< 0.05.

We analyzed the effect of this combination on inhibiting tumor growth and metastasis in two NSCLC xenograft mouse models. Mice with established flank tumors of equal volumes were divided into different treatment groups: DOTAP: cholesterol (DC)- empty vector (EV) complex; DC-TUSC2 complex; erlotinib alone; and DC-TUSC2 complex plus erlotinib. In the H322 subcutaneous xenograft model, the combination of intravenous TUSC2 nanovesicles and erlotinib was significantly superior (P< 0.05) in reducing tumor volumes than either agent alone (Fig 3A). The mean tumor volume was 421.25±89.27 mm^3^, compared with 1082.50±338.69 mm^3^, 801.25±144.60 mm^3^, 675.00±228.80 mm3, and 875.00±267.85 mm^3^, in their counterparts receiving PBS, DC-TUSC2, erlotinib, or EV-erlotinib alone, respectively. In term of tumor size, the posterior probability of cooperative effect was 0.9928, which means that there were less than 100 in 10,000 chances that the effect of TUSC2-erlotinib combination was not cooperative.

A lung metastasis xenograft mouse model was developed using the human TUSC2-defective wild type EGFR A549 NSCLC cell line. Animals were treated with the same protocol as their subcutaneous counterparts. Tumor nodules on lung surfaces were reduced by 82% after TUSC2-erlotinib treatment, compared to 41% and 54%, for TUSC2 or erlotinib treatment, respectively (Fig 3B). The average of number of apoptotic cells in the erlotinib group was 5% (Fig 3C). This value increased to 15% in the group receiving TUSC2-nanovesicles, and reached over 80% in their counterparts receiving TUSC2 and erlotinib. These results show that the growth inhibitory benefit of TUSC2-erlotinib in vitro could be reproduced in vivo and validate the efficacy of this combination.

### Gene Expression Profile and Pathway Analysis Identifies FGFR2 as a Potential Mediator of TUSC2-Erlotinib Cooperative Activity

To understand the molecular basis of TUSC2-erlotinib cooperativity and identify specific pathways involved, we used high-throughput qRT-PCR arrays enabling multi-parallel expression profile analysis of 86 receptor and non-receptor tyrosine kinase genes across TUSC2-deficient wild type EGFR A549, H1299, and H322 NSCLC lines. The TUSC2 expression level served as a positive control. First, the dataset for each cell line was analyzed individually to generate a set of genes whose expression levels were altered between different treatment groups. Then, the resulting aggregate scores were used for pathway analysis based on a false detection rate (FDR) value of 0.05, and fold change (FC) ranging from -2 and +2 fold down- or up-regulated with respect to each agent alone at p < 0.05. For all three cell lines, the TUSC2-erlotinib combination resulted in a common change of expression levels of twelve tyrosine kinase genes. Nine genes, TIE1, ROS1, MATK, EPHA5, ROR2, FGFR3, JAK3, FES, and PTK2B were upregulated, and three genes, PTK2, FLT3, and FGFR2, were downregulated (Table 2). Expression of FGFR2 showed the greatest degree of downregulation by the TUSC2-Erlotinib combination, thus, the specific role of FGFR2 in TUSC2-erlotinib cooperativity was interrogated.

**Table 2:** Gene expression significantly altered in three lung cancer cell lines in the combination group vs erlotinib and empty vector.

Molecules	Fold Change
TUSC2	379.698
TIE1	5.316
ROS1	2.339
MATK	1.967
EPHA5	1.762
ROR2	1.728
FGFR3	1.715
JAK3	1.61
FES	1.583
PTK2B	1.528
PTK2	-0.911
FLT3	-0.555
FGFR2	-0.284

The Fold Change values are log A/log B where A is the gene expression value in the combination group and B is the expression value of the same gene in the erlotinib empty vector group.

### TUSC2-Erlotinib Cooperative Effect Involves FGFR2 Downregulation by TUSC2 in a Cell Specific Manner

**Fig 4:**
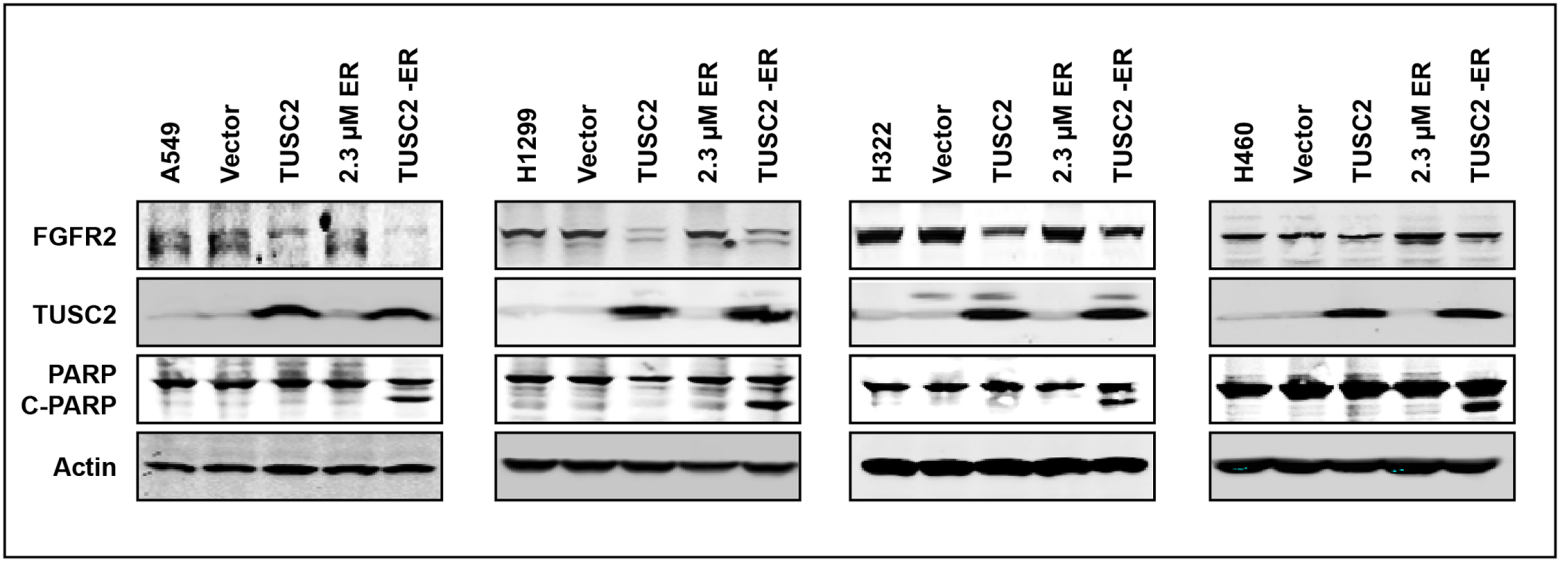
TUSC2 downregulates FGFR2 expression. Cells were transfected with TUSC2 (4μg) expression plasmids with lipofectamine 2000 and treated with erlotinib (2.3 μM). Western blots were performed with the indicated antibodies.

To validate the FGFR2 microarray target data, A549, H1299, H322, and H460 NSCLC cells were transfected with TUSC2 plasmid, treated with 2.3μM erlotinib, and analyzed for FGFR2 expression by western blot. TUSC2 alone significantly reduced FGFR2 expression level in all four cell lines (Fig 4). In contrast, erlotinib alone had no noticeable effect on FGFR2 expression. TUSC2-erlotinib combined treatment inhibited FGFR2 expression to the same extent as that of TUSC2 forced expression alone, and significantly increased levels of PARP cleavage.

**Fig 5:**
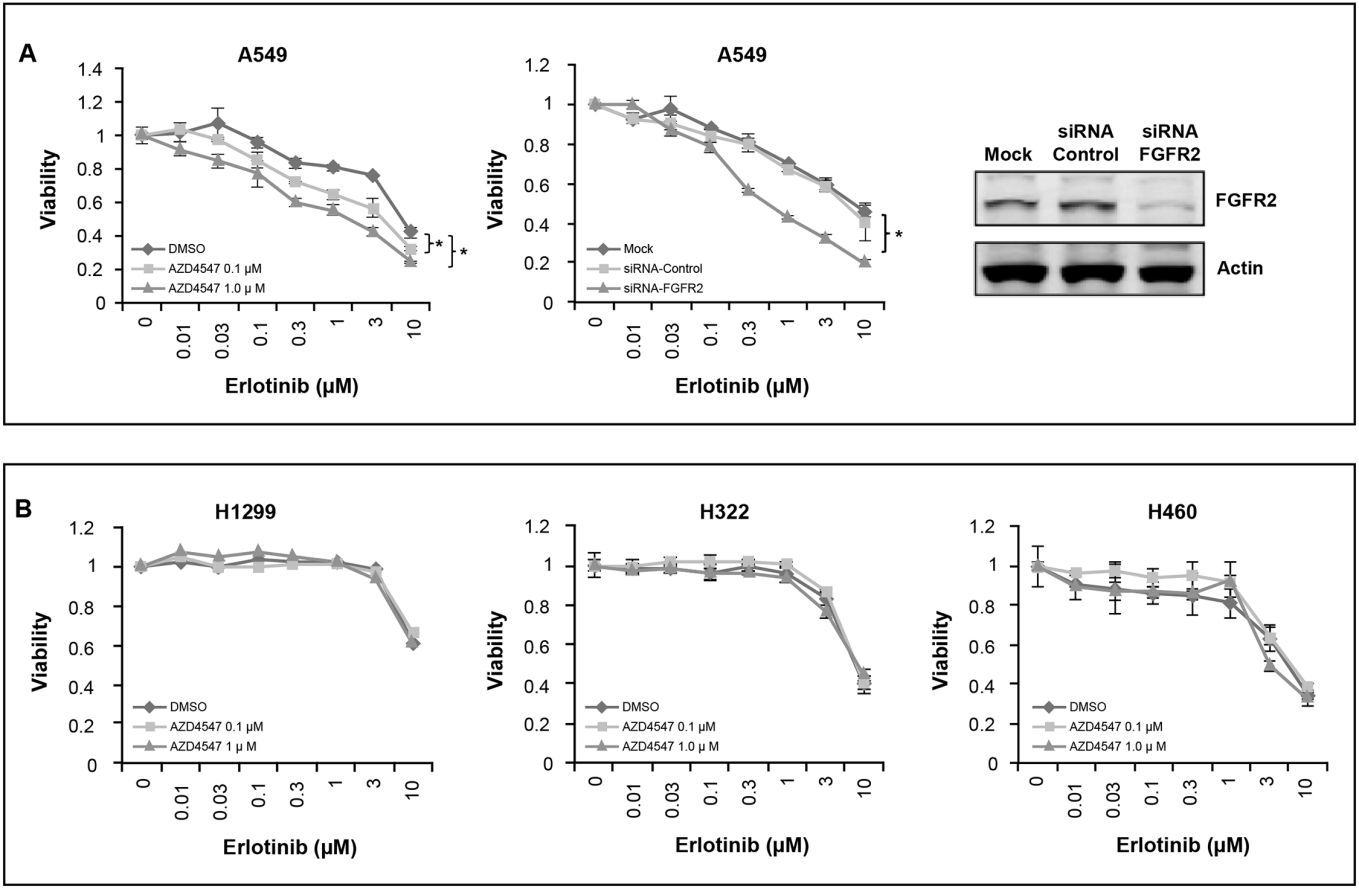
FGFR2 inhibition sensitizes cells to erlotinib in a cell specific manner. Cells were treated with 0.1μM and 1μM FGFR2 inhibitor AZD4547, or transfected with siRNA-FGFR2 construct, exposed to the indicated doses of erlotinib, and assessed for viability using the SRB assay. Real-time PCR analysis of FGFR2 mRNA expression confirmed FGFR2 gene knockdown. Values represent the mean of three independent experiments, each with duplicate samples; *, P< 0.05.

To further understand the role of FGFR2 in TUSC2-enhanced sensitivity to erlotinib, we suppressed FGFR2 expression, by both the selective inhibitor AZD4547 and gene knockdown, and assessed growth and survival after erlotinib exposure. Viability of A549 was significantly reduced (Fig 5A), whereas growth and survival of H1299, H322, and H460 cells were not affected (Fig 5B), suggesting that FGFR2 mediation of the TUSC2-erlotinib cooperative effect is cell context specific.

### Inhibition of mTOR Sensitizes AZD4547 Unresponsive Cells to Erlotinib

**Fig 6:**
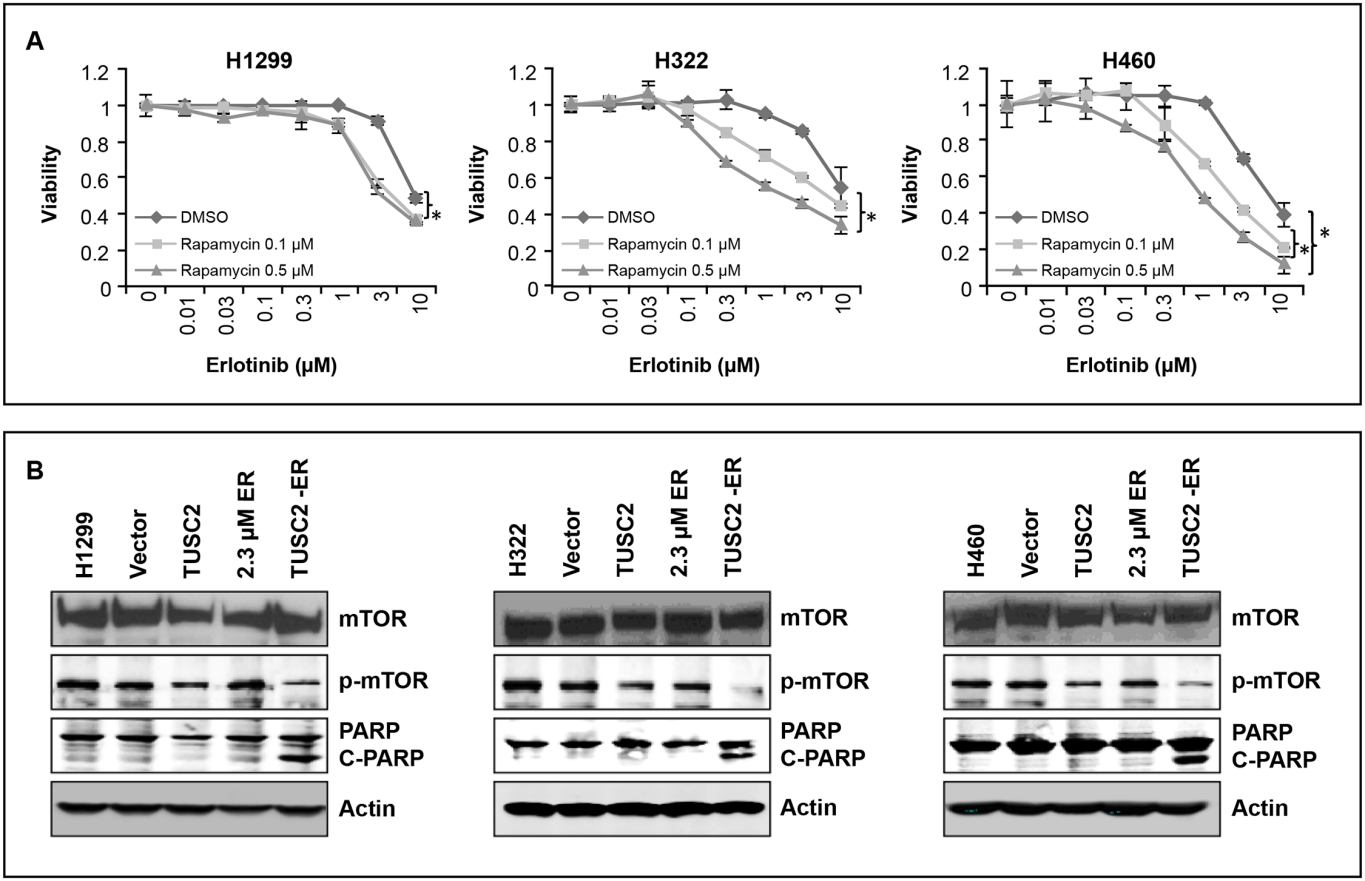
Rapamycin sensitizes AZD4547 unresponsive cells to erlotinib. A) Cells were treated with 0.1μM and 0.5μM mTOR inhibitor Rapamycin, exposed to the indicated dosages of erlotinib, and assessed for viability using the SRB assay. Values represent the mean of three independent experiments, each with duplicate samples; *, P< 0.05. B) Treated cells were lysed and expression of proteins was analyzed by Western blots with the indicated antibodies.

In a prior study, we showed that TUSC2 reduced mTOR phosphorylation and kinase activity in NSCLC cells [19]. Because AZD4547 showed no cooperative effect with erlotinib in H1299, H322, and H460 cells (Fig 5B), we assayed for enhanced erlotinib sensitization by the mTOR inhibitor rapamycin. The results show a pronounced cell death following treatment with rapamycin and erlotinib, starting at nanomolar ranges (Fig 6A). Viabilities of H1299, H322, and H460 were reduced by 50%, 60%, and 80%, respectively. Western blot analysis show that unlike erlotinib, TUSC2 transfection alone downregulated mTOR phosphorylation. Inhibition of the latter was further enhanced after exposure to the TUSC2-erlotinib combination, with a significant increase in PARP cleavage (Fig 6B). These findings suggest that mTOR signaling is involved in the TUSC2-erlotinib cooperative effect in cell lines where cooperativity is not mediated by FGFR2, and indicate an increase in apoptotic activity by the combination.

## Discussion

TUSC2 protein is reduced or absent in over 80% of lung cancers [11, 20]. We previously showed that exogenous expression of TUSC2 induced apoptosis in lung cancer cell lines and inhibited lung tumor xenograft growth and metastasis in mouse models [8, 21]. These findings led to a Phase I clinical trial that showed safety and antitumor activity of TUSC2 nanoparticle-based systemic gene therapy administered intravenously in lung cancer patients [1]. It is not yet possible to restore gene expression with small molecule drugs. Thus, gene replacement is a potentially useful strategy in cancers with tumor suppressor gene loss of function mutations and deletions, which are among the most common genetic abnormalities in all cancers [22]. A possible advantage of combining tumor suppressor gene therapy with small molecule targeted therapy, such as erlotinib, is enhancement of sensitivity that may extend over wild type EGFR cell types with differing mechanisms of drug resistance.

We hypothesized that TUSC2 systemic delivery to wild type EGFR lung cancer cells would complement therapeutic benefits of existing small molecule drugs. In this report, we show that TUSC2 restoration could potentiate sensitivity to the tyrosine kinase inhibitor erlotinib. In vitro, we tested various NSCLC cell types with different genetic backgrounds to rule out the possibility that the effect of TUSC2-erlotinib combination therapy could be cell line or a gene-specific. In vivo, we used two xenograft mouse models, subcutaneous tumor growth and lung metastasis formation, of wild type EGFR NSCLC H322 and A549 cell lines, respectively. As expected, mice tolerated the combination treatment well without any apparent toxicity or adverse effects. Both TUSC2 and erlotinib exhibit low toxicities; therefore, it was likely that the combination would have low toxicity. Tumors were established for 10 to 16 days prior to treatment to provide a more rigorous therapeutic test.

When TUSC2 restoration, through stable or transient forced expression, was combined with erlotinib treatment at drug concentrations clinically attainable, a strong cooperative antitumor effect was observed. Cell growth and colony formation was significantly inhibited in adenocarcinoma and non-adenocarcinoma cell lines. These findings were independent of the technique used to mediate TUSC2 gene expression. TUSC2-erlotinib antitumor efficacy could be reproduced in vivo. Intravenous TUSC2 systemic nanovesicle-based gene delivery and erlotinib treatment significantly reduced xenograft tumor growth and metastasis more effectively than either agent alone, in both mouse models. In addition, in situ TUNNEL assays in resected lung tissues showed a marked increase in the number of tumor cells undergoing apoptosis. In an orthotopic mouse model, the combination of TUSC2 and gefitinib also showed a marked reduction in tumor growth indicating that the effect was not restricted to erlotinib (data not shown). Our results highlight the clinical potential of molecular-based tumor targeting nanoparticles as gene therapy vehicles as well as the therapeutic benefit of TUSC2-erlotinib combination in wild type EGFR NSCLC.

TUSC2-erlotinib antitumor benefit in cells without EGFR mutations suggests that TUSC2 regulates pathways other than EGFR that render cells more sensitive to erlotinib. Thus, we interrogated the ability of other kinase pathways to regulate this cooperative effect. mRNA expression profiling of eighty six receptor and non-receptor tyrosine kinase genes in three TUSC2-deficient wild type EGFR cell lines, transfected with TUSC2 and treated with erlotinib, revealed consistent changes in 12 genes. Expression levels of five receptor and four non-receptor molecules were increased, whereas, those of two receptor and one non-receptor molecules were reduced, indicating regulation of the TUSC2-erlotinib cooperative effect by multiple tyrosine kinase pathways. We focused on FGFR2 expression because its level was reduced to the greatest extent. In addition, a previous study has highlighted FGFR2 signaling as a mechanism of acquired resistance to EGFR TKIs, and suggested that treatment of NSCLC patients with combinations of EGFR and FGFR specific TKIs may be a strategy to enhance efficacy of single EGFR inhibitors [23]. We found a significant inhibition of FGFR2 expression by TUSC2 transient transfection alone, suggesting a functional role for TUSC2 in the observed cooperative effect. Erlotinib had no effect and the TUSC2-erlotinib combination showed a similar effect as TUSC2 alone. There was a marked increase of PARP cleavage levels in all tested cells, indicating enhanced apoptotic activity by the combined treatment.

Suppression of FGFR2 with the selective inhibitor, AZD4547, or siRNA directed against FGFR2, sensitized one cell line to erlotinib, but had no apparent effect in three other cell lines with both high and low levels of FGFR2 expression. One plausible explanation for this finding is that while FGFR2 downregulation might be implicated in TUSC2-induced sensitivity of A549 cells to erlotinib, other pathways mediate this cooperative effect in H1299, H322, and H460 cells. We have previously reported a significant role for TUSC2 in sensitizing NSCLC to the AKT inhibitor MK2206, a cooperative effect involving inhibition of mTOR phosphorylation and enzymatic activity [20]. Thus, implication of mTOR in TUC2-erlotinib efficacy was interrogated in cells not responsive to FGFR2 and EGFR inactivation. As expected, these cells were very responsive to rapamycin and erlotinib combined treatment, which inhibited tumor cell growth and viability cooperatively. Thus, TUSC2-erlotinib cooperation cannot be explained by perturbations in a single common pathway. TUSC2 is acting as a multifunctional protein, and its mechanism of interaction with erlotinib appears to be dependent on the cellular context and involves pathways other than EGFR. Further support for this notion came from a study describing a synergistic interaction between rapamycin, an mTOR inhibitor, and erlotinib in non-small cell lung cancer cell lines [24].

The DC nanovesicles used to deliver TUSC2 in vivo can achieve gene delivery at therapeutic levels. Other lipid formulations have been ineffective because of instability *in vivo*, due in part to interactions with serum proteins [25], Templeton and coworkers showed that DC nanovesicles were more efficient for gene delivery system than other lipid formulations [26]. Uptake by the liver and spleen was greatly reduced and distribution to other organs was increased, with the lung showing the highest level of gene expression. The DC formulation prevents protein binding to nanovesicles which reduces clearance by the reticuloendothelial system [27, 28]. Cationic lipids enhance nucleic acid delivery by increasing the efficiency of nucleic acid transfer. The addition of neutral lipids to the cationic lipids increases nanoparticle rigidity and stability [29]. The DC lipoplex formulation was used for our study because it achieves a balance of low toxicity and efficient nucleic acid transfer in vivo and has proven activity and safety in a clinical trial [1, 26]. Uptake of nanovesicles by tumor cells was 10-fold greater than that of normal cells because of enhanced endocytosis by tumor cells, thus imparting a passive targeting property without the need to attach ligands [30]. The plasmid was optimized by using a high-copy number pMB1 plasmid replication origin element, incorporating a mini-CMV promoter which is not tumor selective, and removing unnecessary plasmid backbone sequences to minimize size. Additional modifications included adding E1 enhancer elements with the mini-CMV promoter which increases the expression of the transgene [31, 32].

DC TUSC2 nanovesicles were safely administered in a phase 1 clinical trial in patients with advanced stage of lung cancer, previously treated with platinum-based chemotherapy.[1]. The study showed that DOTAP:chol-TUSC2 can be safely administered intravenously in lung cancer patients and results in: 1) uptake of the gene by human primary and metastatic tumors; 2) transgene and gene product expression at therapeutic levels; 3) specific alterations in TUSC2-regulated pathways; and 4) anti-tumor effects, which is the first time this has been reported for systemic DOTAP:cholesterol nanovesicle gene therapy. Thus it is feasible to use DC TUSC2 nanovesicles in combination with erlotinib, and a clinical trial with combination has been initiated.

In conclusion, we present compelling biological evidence demonstrating a functional capacity of TUSC2 gene restoration to sensitize wild type EGFR NSCLC cells to erlotinib treatment, in vitro and in vivo. Neither treatment alone was able to reduce tumor cell death and growth as effectively as the combination. However, this activity is dependent on the cellular context and involves inactivation of pathways other than EGFR, which we expected, since we did not target mutated EGFR. Our findings provide new insights into the molecular mechanisms of TUSC2-mediated tumor suppression, and suggest that the therapeutic activity of TUSC2 could extend the use of erlotinib to lung cancer patients with wildtype EGFR.

## Supporting Information

S1 Table Tyrosine Kinase Genes (86). (DOCX)
