# Supplementary material for: Exogenous Restoration of TUSC2 Expression Induces Responsiveness to Erlotinib in Wildtype Epidermal Growth Factor Receptor (EGFR) Lung Cancer Cells through Context Specific Pathways Resulting in Enhanced Therapeutic Efficacy
Source: PLoS One. 2015 Jun 8;10(6):e0123967. doi: 10.1371/journal.pone.0123967 (PMC4460038; doi:10.1371/journal.pone.0123967)
Supplement: S1 Table — (DOCX) [file pone.0123967.s001.docx]

**TABLE S1: Tyrosine Kinase Genes (86)**

| ABL1, ABL2, ALK, AXL, BLK, BTK, CSF1R, CSK, DDR1, DDr2, EGFR, EPHA1, EPHA2, EPHA3 EPHA4, EPHA5, EPHA7, EPHA8, EPHB1, EPHB2, EPHB3, EPHB4, EPHB6, ERBB2, ERBB3, ERBB4 FER, FES, FGFR1, FGFR2, FGFR3, FGFR4, FGR, FLT1, FLT3, FLT4, FRK, FYN, HCK, IGF1R, IGF2R, INSR, INSRR, ITK, JAK1, JAK2, JAK3, KDR, KIT, LCK, LTR, LYN, MATK, MERTK, MET MST1R, MUSK, NTRK1, NTRK2, NTRK3, PDGFRA, PDGFRB, PTK2, PTK2B, PTK6, PTK7, RET, ROR1, ROR2 ROS1, RYK, SRMS, SRC, SYK, TEC, TEK, TIE1, TNK1, TNK2, TXK, TYK2, TYRO3, YES1, ZAP70, p53, TUSC2, **GAPDH** |
| --- |
